# Psychometric Properties of the Nine‐Item Personal Health Questionnaire (PHQ‐9) Seven‐Item Generalised Anxiety Disorder Scale (GAD‐7), and the Work and Social Adjustment Scale (WSAS) With People With Intellectual Disabilities

**DOI:** 10.1111/jir.13231

**Published:** 2025-03-17

**Authors:** Dave Dagnan, Rob Saunders, Joshua Stott, Richard Thwaites, Chris Hatton

**Affiliations:** ^1^ Cumbria, Northumberland, Tyne & Wear NHS Foundation Trust Workington UK; ^2^ CORE Data Lab, Research Department of Clinical, Educational and Health Psychology UCL London UK; ^3^ ADAPT Lab, Research Department of Clinical, Educational and Health Psychology UCL London UK; ^4^ Cumbria, Northumberland, Tyne and Wear NHS Foundation Trust Penrith UK; ^5^ Manchester Metropolitan University Manchester UK

**Keywords:** intellectual disability, mental health, psychiatric disorders, treatment and services

## Abstract

**Background:**

The nine‐item Physical Health Questionnaire (PHQ‐9), the seven‐item Generalised Anxiety Disorder scale (GAD‐7) and the Work and Social Adjustment Scale (WSAS) are, respectively, self‐report measures of depression, generalised anxiety, and the impact of mental health on the person's personal functioning that are widely used in mainstream mental health services in England. The psychometric properties of these scales when used with people with intellectual disabilities have not been established.

**Method:**

Item level data for the PHQ‐9 (*n* = 128), GAD‐7 (*n* = 124) and WSAS (*n* = 133) for people with intellectual disabilities in an English NHS Talking Therapies for anxiety and depression (NHSTT) service in the north of England were analysed using internal reliability statistics and confirmatory factor analysis.

**Results:**

In this study, the full PHQ‐9, GAD‐7 and WSAS have Cronbach's *α* of 0.81, 0.84 and 0.81, respectively, and have acceptable ranges of corrected item‐total correlations. The two‐factor structures for the PHQ‐9 and the GAD‐7 were a better fit than single‐factor structures, although the single‐factor fit and the correlation between the two factors within each scale suggest that their use as a single scale is justified. The single‐factor structure for the WSAS was a good fit.

**Conclusions:**

In this study, the widely used PHQ‐9, GAD‐7 and WSAS demonstrate internal consistency values and factor analysis structure similar to those for individuals without intellectual disabilities. The data support the use of these measures for people with intellectual disabilities attending routine primary care mental health services.

## 1 Introduction

Anxiety and depression are at least as prevalent for people with intellectual disabilities as for people who do not have intellectual disabilities. Point prevalence for anxiety disorders has been reported as 3.8% of adults with intellectual disabilities (Reid et al. 2011) and as 6.5% for unipolar depression (Cooper et al. 2018). Anxiety and depression are associated with numerous adverse consequences, including a higher likelihood of challenging behaviour, loneliness and sleep disorder (e.g., Hurley 2008; Bond et al. 2020). Systematic reviews have found that psychological therapies can be effective for people with intellectual disabilities and some mental health presentations. For example, Tapp et al. (2023) found the strongest effects were for treatments of anger and that treatments of anxiety and depression had small and not significant effects. Better understanding of the properties of widely used measures of mental ill‐health when used with people with intellectual disabilities will support the further development of evidence in this area.

Self‐report measures of depression and anxiety, such as the 20‐item Glasgow Depression Scale (GDS; Cuthill et al. 2003) and the 27‐item Glasgow Anxiety Scale for People with Intellectual Disabilities (GAS‐ID; Mindham and Espie 2003), have been developed specifically for use with people with intellectual disabilities. Such scales are typically developed through working with people with intellectual disabilities to develop accessible presentation and wording of scale items whilst maintaining diagnostically relevant questions (Cuthill et al. 2003; Mindham and Espie 2003). These measures offer a useful tool in specialist services for people with intellectual disabilities. However, they are relatively long, can take a considerable part of a clinical contact to deliver and do not allow comparison of outcomes with people who do not have intellectual disabilities.

Measures designed for mainstream populations that are used with people with intellectual disabilities are less commonly described. However, when such scales are used with people with intellectual disabilities, they are often found to have similar properties to when they are used with people without intellectual disabilities; for example, Wieland et al.'s (2012) analysis of a Dutch translation of the 53 item ‘Brief Symptom Inventory’ (Derogatis 1993) and Powell's (2003) and Ailey's (2008) analysis of the BDI‐II (Beck et al. 1996). Some of the most widely used measures in mainstream English mental health services are the seven‐item Generalized Anxiety Disorder scale (GAD‐7; Spitzer et al. 2006), the nine‐item depression scale, the Patient Health Questionnaire (PHQ9; Kroenke et al. 2001) and the five‐item Work and Social Adjustment Scale (WSAS; Mundt et al. 2002), which measures the impact of mental health problems on social functioning. The PHQ‐9 is one of the UK NICE guidance recommended self‐report assessments for depression (NICE 2024) and has a particular advantage in NHS services, as it is both brief and free to use. The GAD‐7 is recommended in NICE guidance for assessing symptoms of generalised anxiety (NICE 2020) and is also brief and free to use. The PHQ‐9 and the GAD‐7 are part of the core data set used in NHS Talking Therapies for anxiety and depression (NHSTT) in England. The Work and Social Adjustment Scale (Mundt et al. 2002; Zahra et al. 2014) is a five‐item self‐report scale that measures functional impairments associated with a named problem area. It is also part of the core data set for NHSTT and has been shown to measure a distinct social functioning factor (when used alongside the PHQ‐9 and the GAD‐7; Zahra et al. 2014).

The psychometric properties of these scales are very well reported (see discussion in Section 2 of this paper), and it is important that the characteristics of the PHQ‐9, GAD‐7 and WSAS, when used with people with intellectual disabilities, are understood to support comparison of treatment outcomes for people with intellectual disabilities and those without intellectual disabilities in services such as NHSTT. These services treat very large numbers of people (in 2023–2024 NHSTT in England received 1.83 million referrals; NHS Digital 2024), of which a proportion can be identified as people with intellectual disabilities. NHSTT produces large amounts of routine data with outcomes that are based on the known properties of mandated scales, including the PHQ‐9, GAD‐7 and WSAS. A small number of papers have begun to report outcomes for people with intellectual disabilities compared with those who do not have such disabilities using national data sets (e.g., Dagnan et al. 2022; El Baou et al. submitted for publication). Fully understanding the properties of the scales when used with people with intellectual disabilities will give greater confidence to the findings in such studies.

The GAD‐7 and the PHQ‐9 have an additional interest in that Kroenke et al. (2009) provided evidence for a brief screening scale, the PHQ‐4, which was based on the first two items from each of the PHQ‐9 and GAD‐7; from the PHQ‐9 (‘Feeling down, depressed or hopeless’ and ‘Little interest or pleasure in doing things’) and GAD‐2 (‘Feeling, nervous anxious or on edge’ and ‘Not being able to stop or control worrying’). The psychometric and normative data for these scales have been recently updated (Wicke et al. 2022) and are generally satisfactory. Self‐report measures for people with intellectual disabilities tend to take longer to administer than for people without intellectual disabilities and brief screening measures that can be used in mainstream and specialist services would be of considerable value in better identifying people with intellectual disabilities with depression and anxiety.

The primary aim of this paper is to report confirmatory factor analysis (CFA), internal reliability and between scale correlations for the PHQ‐9, the GAD‐7 and the WSAS when used with people with intellectual disabilities. A secondary aim is to report properties of the subset of items that constitute the PHQ‐4.

## 2 Method

### 2.1 Ethics and information governance.

The data were obtained in 2019 and consisted of all people who had been referred to a northern English NHSTT service in the period 2009–2019. The NHSTT data were linked to the coterminous GP record, and a code was generated indicating the people who were on the GP register for people with intellectual disabilities (SNOMED code 416075005, ‘on learning disability register’; NHS England and NHS Improvement 2019); these data were then fully anonymised. In November 2019, the data were extracted, linked and anonymised for the purpose of evaluation and service improvement, and this was the legal basis for processing the data. Information Governance and Research and Development systems confirmed that the data were regarded as appropriately anonymised for their original purpose and their subsequent use in this research project and were out of scope of General Data Protection Regulations (GDPR; Information Commissioners Office 2023).

The routine collection data at every clinical and assessment session are described in the NHSTT manual (National Collaborating Centre for Mental Health 2024). The collection of PHQ‐9, GAD‐7 and WSAS total scores is required at every session, and typically, a total score is calculated and entered by the therapist. During the period that the current data were collected, therapists had an option to enter item levels scores; this option was not taken up by all therapists.

### 2.2 Participants

The total data set available consisted of 93 527 referrals (35685 [38.2%] male, mean age 39.4 years [SD = 15.9]) of which 423 referrals were identified as people with intellectual disability (0.45%; 226 [53.4%] male, mean age 31.4 years [SD = 12.0]); of these 128 referrals had all items available for the PHQ‐9 (64 [51.6%] male, mean age 29.2 years [SD = 10.8]) and 133 (66 [49.6%] male, mean age 26.7 years [SD = 10.1]) and 124 had all items for the GAD‐7 (62 [50.0%] male, mean age 29.1 years [SD = 10.8]) and 133 (66 [49.6%] male, mean age 26.7 years [SD = 10.1]). It is possible that characteristics of people's presentation influenced whether item level data were recorded. However, there was no significant difference in PHQ‐9 means between those who only had total score data and those who had item level data [total score only group mean = 14.45, SD = 6.10, *n* = 164; item level data group mean = 14.02, SD = 6.39, *n* = 128; t = 0.58, df = 290, ns]. There was no significant difference in GAD‐7 means between those who only had total score data and those who had item level data [total score only group mean = 12.98, SD = 5.32, *n* = 171; item level data group mean = 12.72, SD = 5.46, *n* = 124; t = 0.41, df = 293, ns]. In this paper, we have used a four‐item version of the WSAS; thus, we do not present comparisons as the total scores in the full data set have not been calculated from only four items.

### 2.3 Measures

#### 2.3.1 Patient Health Questionnaire‐9 (PHQ‐9, Kroenke et al. 2001)

The psychometric properties of the PHQ‐9 are well reported (e.g., Patel et al. 2019; Vu et al. 2022). For example, Boothroyd et al. (2019) used data from the same service as the current study. They used Mokken analysis and CFA and found that a two‐factor model (somatic and cognitive‐affective) had a better fit than a single‐factor model. The two‐factor structure found by Boothroyd et al. (2019) identified one factor containing cognitive and affective symptoms and the second factor containing somatic symptoms; the cognitive‐affective items loading on factor 1 were items 1 (Anhedonia), 2 (Depressed mood), 6 (Feelings of worthlessness) and 9 (Suicidal ideation). The somatic factor included Items 3 (Sleep difficulties), 4 (Fatigue), 5 (Appetite changes), 7 (Concentration difficulties) and 8 (Psychomotor agitation). Boothroyd et al. (2019) report the full scale PHQ‐9 as having a Cronbach's *α* of 0.90, the cognitive items an *α* of 0.86 and the somatic items an *α* of 0.83. They also suggest that the correlation of the two factors and the data from the single‐factor solution show that the PHQ‐9 total score is also acceptable (see also; Bianchi et al. 2022).

#### 2.3.2 Generalised Anxiety Disorder‐7 (GAD‐7, Spitzer et al. 2006)

The psychometric properties of the GAD‐7 are also well known. Beard and Björgvinsson (2014) found a two‐factor structure accounted for 70% of the variance; the first factor included items 1 (Feeling nervous, anxious or on edge), 2 (Not being able to stop or control worrying), 3 (Worrying too much about different things) and 7 (Feeling afraid as if something awful might happen). The second factor included the remaining items 4 (Trouble relaxing), 5 (Being so restless that it is hard to sit still) and 6 (Becoming easily annoyed or irritable). This two‐factor structure is supported by Boothroyd et al. (2018) and suggests a cognitive and somatic/behaviour structure that parallels that of the PHQ‐9. Cronbach's *α* for the full GAD‐7 is reported as 0.81 (Johnson et al. 2019), the cognitive items have an *α* of 0.84 and the somatic items have an *α* of 0.72 (Boothroyd et al. 2018). Similarly to the PHQ‐9, there is also evidence to support the use of the scale as single factor (e.g., Johnson et al. 2019).

#### 2.3.3 Work and Social Adjustment Scale (WSAS, Mundt et al. 2002)

Zahra et al. (2014) reported that the WSAS had a Cronbach's *α* of 0.82 and a single‐factor structure that was distinct from the PHQ‐9 and GAD‐7. In this paper, the WSAS item relating to the impact of wellbeing on work functioning is not used, as only 33 (24.8%) of the people with intellectual disabilities who supplied item level data were employed and therefore could provide an answer to this item. The use of the WSAS as a four‐item measure has not previously been reported; however, it is expected that a subset of the five‐item single‐factor WSAS will also have a single‐factor structure, and any subdivision of this scale would produce unacceptably small subscales (Boateng et al. 2018).

### 2.4 Analysis

Based upon the extensive previous research on the structure of the PHQ‐9 and GAD‐7, CFA was carried out to test the fit of both single‐factor models and models that split the two scales into somatic and cognitive/affective scales. CFA was carried out to test the fit of a single‐factor structure for the four‐item WSAS.

Cronbach's *α*, item means, standard deviations, corrected item total correlations for each scale and correlations between scales (Pearson's *r*) were calculated using SPSS 24. CFA was carried out with the Lavaan package in R (Rosseel 2012) using the maximum likelihood estimator as the data are continuous. The comparative fit index (CFI), the Tucker–Lewis index (TFI), the standardized root mean squared residual (SRMR) and the root mean square error of approximation (RMSEA) were used as measures of fit. We considered CFI and TFI scores of ≥ 0.95 as indicative of good model fit and scores of < 0.08 and 0.07 as representing good fit on the SRMR and RMSEA, respectively (Hooper et al. 2008).

## 3 Results

### 3.1 Scale analysis

Mean total PHQ‐9 scores were 14.02 (SD = 6.39, *n* = 128), mean total GAD‐7 scores were 12.72 (SD = 5.46, *n* = 124) and the four‐item WSAS mean scores were 11.96 (SD = 8.47, *n* = 133). Tables 1, 2 and 3 show item mean, standard deviation and corrected item‐total correlation for each of the three scales; none of the item‐total correlations for any of the scales are outside acceptable ranges (Penfield 2013). In this study, the full PHQ‐9 has a Cronbach's *α* of 0.81, the full GAD‐7 has a Cronbach's *α* of 0.84, and the four‐item WSAS has a Cronbach's *α* of 0.81.

**TABLE 1:** Item means, standard deviations and corrected item‐total correlations for the PHQ‐9 (*n* = 124).

Patient Health Questionnaire‐9 Items Over the last 2 weeks, how often have you been bothered by any of the following problems?	Item mean	Item standard deviation	Corrected item‐total correlation
1. Little interest or pleasure in doing things	1.60	1.14	0.57
2. Feeling down, depressed, or hopeless	2.02	0.95	0.59
3. Trouble falling or staying asleep, or sleeping too much	1.85	1.18	0.53
4. Feeling tired or having little energy	1.80	1.15	0.50
5. Poor appetite or overeating	1.45	1.22	0.56
6. Feeling bad about yourself—or that you are a failure or have let yourself or your family down	1.78	1.18	0.48
7. Trouble concentrating on things, such as reading the newspaper or watching television	1.52	1.20	0.52
8. Moving or speaking so slowly that other people could have noticed? Or the opposite—being so fidgety or restless that you have been moving around a lot more than usual.	1.16	1.11	0.43
9. Thoughts that you would be better off dead or of hurting yourself in some way.	0.82	0.98	0.37

**TABLE 2:** Item means, standard deviations and corrected item‐total correlations for the GAD‐7 (*n* = 124).

Generalised Anxiety Disorder‐7 Items	Item mean	Item standard deviation	Corrected item‐total correlation
Over the last 2 weeks, how often have you been bothered by any of the following problems?			
1. Feeling nervous, anxious or on edge	2.02	1.01	0.57
2. Not being able to stop or control worrying	2.06	1.03	0.74
3. Worrying too much about different things	2.02	1.02	0.60
4. Trouble relaxing	1.74	1.16	0.65
5. Being so restless that it is hard to sit still	1.19	1.18	0.56
6. Becoming easily annoyed or irritable	1.97	0.99	0.46
7. Feeling afraid as if something awful might happen	1.72	1.17	0.53

**TABLE 3:** Item means, standard deviations and corrected item‐total correlations for the WSAS (*n* = 133).

Work and Social Adjustment Scale Items‐4 items	Item mean	Item standard deviation	Corrected item‐total correlation
1. Because of my [problem] my home management (cleaning, tidying, shopping, cooking, looking after home or children, paying bills) is impaired.	2.68	2.48	0.64
2. Because of my [problem] my social leisure activities (with other people e.g. parties, bars, clubs, outings, visits, dating, home entertaining) are impaired.	3.19	2.88	0.71
3. Because of my [problem], my private leisure activities (done alone, such as reading, gardening, collecting, sewing, walking alone) are impaired.	2.71	2.60	0.61
4. Because of my [problem], my ability to form and maintain close relationships with others, including those I live with, is impaired.	3.38	2.55	0.60

### 3.2 Confirmatory factor analysis

Table 4 shows data from the single and two‐factor models for the PHQ‐9 and the GAD‐7 and the single‐factor model for the WSAS. For the PHQ‐9, the two‐factor model was a slightly better fit, the two PHQ‐9 factors were highly correlated (*r* = 0.79, *p* > 0.001), the cognitive factor had Cronbach's *α* of 0.70, and the somatic factor has Cronbach's *α* of 0.74. For the GAD‐7, the two‐factor model was a marginally better fit, the two factors were highly correlated (*r* = 0.86, *p* < 0.001), the cognitive factor had a Cronbach's *α* of 0.70, and the somatic factor had a Cronbach's *α* of 0.67. The single‐factor structure for the four‐item WSAS indicates a good level of fit.

**TABLE 4:** Confirmatory factor analysis fit statistics for one‐ and two‐factor models for the PHQ‐9 and GAD‐7 and one factor model for the WSAS.

PHQ‐9 (*n* = 124), GAD‐7 (*n* = 124) and WSAS (*n* = 133) models	*χ* ^2^	df	CFI	TLI	RMSEA (90% CI)	SRMR
PHQ‐9 single‐factor model	51.4**	27	0.91	0.88	0.083 (0.047–0.117)	0.06
PHQ‐9 two‐factor model	39.4*	26	0.95	0.93	0.063 (0.010–0.100)	0.05
GAD‐7 single‐factor model	47.6**	14	0.89	0.84	0.138 (0.096–0.182)	0.06
GAD‐7 two‐factor model	42.4**	13	0.90	0.85	0.134 (0.090–0.184)	0.06
WSAS single‐factor model	3.0	2	0.99	0.98	0.060 (0.001–0.191)	0.02

* Statistically significant *p* < 0.05.

** Statistically significant *p* < 0.01.

### 3.3 Further analysis

Using the data from those who had item level scores on the PHQ‐9 and the GAD‐7, PHQ‐4 scores were calculated (the summed scores from items one and two from the PHQ‐9 and items one and two from the GAD‐7). The PHQ‐4 had a mean of 7.70 (SD = 3.15, *n* = 122) and Cronbach's *α* for the scale is 0.76. Pearson's correlation between the full scale PHQ‐9, GAD‐7, the four‐item WSAS and the PHQ‐4 were calculated. The PHQ‐4 correlated with the PHQ‐9 (*r* = 0.78, df = 119, *p* < 0.001), the GAD‐7 (*r* = 0.79, df = 120, *p* < 0.001) and the four‐item WSAS (*r* = 0.33, df = 99, *p* < 0.001); the PHQ‐9 correlated with the GAD‐7 (*r* = 0.69, df = 119, *p* < 0.001) and the four‐item WSAS (*r* = 0.50, df = 103, *p* < 0.001); the GAD‐7 correlated with the four‐item WSAS (*r* = 0.33, df = 101, *p* < 0.001).

## 4 Discussion

This paper has described CFA and internal reliability data for the PHQ‐9, GAD‐7 and WSAS when used with people with intellectual disabilities seeking psychological treatment for depression and anxiety disorders. The data were from an NHS Talking Therapies service for anxiety and depression (NHSTT) service with people with intellectual disabilities identified by SNOMED codes from coterminous general practice. The PHQ‐9, GAD‐7 and the four‐item WSAS all have Cronbach's *α* of above 0.80, but below 0.90, which are generally regarded as good internal reliability (Taber 2018). The scales have been well described in people without intellectual disabilities, and this study has used confirmatory analysis to examine the data fit to scale structures previously reported. Confirmatory analysis suggests a broadly acceptable fit for the single‐factor structures for both the PHQ‐9 and GAD‐7 and an improved fit for the two‐factor solution of the PHQ‐9 and a marginally improved fit for the two‐factor solution for the GAD‐7 (Hooper et al. 2008). Similar results are reported for these scales when used with people without intellectual disabilities. For example, Boothroyd et al. (2018), using data from the same service, describe analysis of the PHQ‐9 and report an RMSEA of 0.083 and a CFI of 0.936 and for the two‐factor structure an RMSEA of 0.063 and a CFI of 0.993; these fit statistics are very similar to those in the current study. Boothroyd et al. (2018) describe analysis of the GAD‐7 and for the single‐factor structure report an RMSEA of 0.103 and a CFI of 0.992 and for the two‐factor structure an RMSEA of 0.047 and a CFI of 0.998; these fit statistics are similar to those of the current study, although in the Boothroyd et al. (2018) data, the two‐factor structure is more clearly improved over the single‐factor structure. Hooper et al. (2008) argue that the interpretation of goodness of fit statistics should consider the strength of the model being tested, and in this instance, the results of the current analysis are very similar to structure to those of several previous analyses with larger data sets of people without intellectual disabilities (e.g., Boothroyd et al. 2018; Beard and Björgvinsson 2014). Both the current study and Boothroyd et al. (2018) found that the scales from the two factor solutions, for both the PHQ‐9 and the GAD‐7, were highly correlated, suggesting that a single‐factor structure is an acceptable representation of the scales. The WSAS was found to have a clear single‐factor structure as found when used as a five‐item measure with people without intellectual disabilities (e.g., Zahra et al. 2014). The four‐item WSAS has potential as a measure of the impact of mental health difficulties on the lives of people with intellectual disabilities in both clinical and research contexts.

The scales have internal reliability that is comparable with other studies with people who do not have intellectual disabilities. Boothroyd et al. (2019) report the full scale PHQ‐9 has a Cronbach's *α* of 0.90, the cognitive items an *α* of 0.86 and the somatic items an *α* of 0.83. These values are marginally higher than those found in the current study. For the GAD‐,7 Cronbach's *α* for the full scale is reported as 0.81 (Johnson et al. 2019), the cognitive items have a Cronbach's *α* of 0.84 and the somatic items a Cronbach's *α* of 0.72 (Boothroyd et al. 2018). These values are similar to those in the current study. The four‐item WSAS has not been previously reported, but Zahra et al. (2014) reported that the five‐item WSAS had an alpha of 0.82; in this study, the four‐item WSAS has an alpha of 0.81. Where possible, we have reported comparison data from the studies of Boothroyd et al. (2018, 2019) as these are from the same service population as the data in the current study.

Exploratory correlations are also reported for the PHQ‐9, the GAD‐7, the WSAS and the four‐item PHQ‐4. The initial associations of the PHQ‐4 with the GAD‐7, PHQ‐9 and WSAS for people with intellectual disabilities are similar to those previously reported data for large populations of people without intellectual disabilities. For example, Boothroyd et al. (2018) reported that the PHQ‐4 correlates with the PHQ‐9, the GAD‐7 and the WSAS at 0.81, 0.82 and 0.51, the PHQ‐9 correlates with the GAD‐7 and WSAS scale at 0.65 and 0.56, and the GAD‐7 correlates with the WSAS at 0.44. These correlations compare well with those from the current study, although the WSAS used by Boothroyd et al. (2018) included the work item. The association of the PHQ‐9 and GAD‐7 reported here and in other studies (e.g., Spitzer et al. 2006) is typical of associations between anxiety and depression measures both for people with intellectual disabilities and those without (e.g., Dagnan et al. 2008). There are several theories to explain the strong association between these presentations; for example, the tripartite model (Clark and Watson 1991) has received considerable research attention and suggests that anxiety and depression share a common component of negative affect but can be differentiated by low positive affect associated with depression and high physiological arousal associated with anxiety.

Typically, self‐report scales developed specifically for people with intellectual disabilities have been longer than the PHQ‐9 and the GAD‐7; for example, the Glasgow Depression Scale (Cuthill et al. 2003) and the Glasgow Anxiety Scale (Mindham and Espie 2003) have 20 and 27 items, respectively. Reliable and valid short assessments of depression and anxiety such as the PHQ‐4 that can be repeatedly administered without taking up a large part of available clinical time are potentially very useful, although considerable further research is needed to establish how well they predict the results of more detailed questionnaire and clinical assessments.

The GAD‐7, PHQ‐9 and WSAS are amongst the most widely used assessments of wellbeing in the English National Health Service. In the United Kingdom, national strategy encourages mainstream services to work with people with intellectual disabilities (e.g., McConkey et al. 2020), and there is a requirement to ensure that there is equity of outcome for this group. It is important that, where possible, people with intellectual disabilities and people without intellectual disabilities use the same measures to allow comparison of outcomes. For example, in England, primary care mental health services for people with anxiety and depression are provided through NHS Talking Therapies Services for Anxiety and Depression (NHSTT). These services have several characteristics, but a particular feature is that routine measures are taken at every session and services are judged based on ‘recovery data’ (the percentage of people who start therapy above the caseness cut‐off for depression or anxiety, who go on to finish therapy below the caseness cut‐off for both depression and anxiety; National Collaborating Centre for Mental Health 2024). Studies have begun to compare outcomes for people with and without intellectual disabilities in these services. For example, Dagnan et al. (2022) use nationally reported data to demonstrate that people with intellectual disabilities have poorer outcomes on criteria using the PHQ‐9 and the GAD‐7 than people without intellectual disabilities, although this paper used service level data and adjustment to account for specific demographic features of people with intellectual disabilities was not possible.

This is the first paper to report properties of the PHQ‐9, GAD‐7 and WSAS for people with intellectual disabilities. Based on the results of the current paper, we suggest that work is required to establish further psychometric characteristics of the scales when used with people with intellectual disability. For example, characteristics such as their scalar properties, factor stability, sensitivity to change and test‐retest reliabilities, construct validity compared with other depression and anxiety scales for people with intellectual disabilities and discriminant validity based on psychiatric diagnosis should all be established (Boothroyd et al. 2019; Boateng et al. 2018). However, the results of this paper suggest that there is potential to use these measures in their original format with people with intellectual disabilities. Guidance on their delivery to people with intellectual disabilities is therefore important (e.g., Dagnan et al. 2015). For example, where items have multiple elements, each element can be presented separately and where the response format is challenging for an individual then analogue presentation or a paired approach to response scales can be used (e.g., Dagnan et al. 2015; Cuthill et al. 2003). Research on the adaptation of measures for people with intellectual disabilities has often focussed on changing item wording and focus. However, in the context of services such as NHSTT, changing the wording of items and the associated impact on psychometric properties will mean that establishing equality of outcome for people with intellectual disabilities is not possible. The current paper suggests that further exploration of how to make existing scales more accessible through careful presentation of items might instead be researched.

This study has limitations. The number of participants is small, although similar types of analysis have been reported with similar participant numbers in other studies (e.g., Scior et al. 2023). The results should be interpreted with caution, as smaller samples can lead to an increased risk of model misspecification; however, the factor models tested are very well established, and the similarity of the other data characteristics to studies with larger population of people without intellectual disability suggests that the current data are a good representation of how the scales perform for people with intellectual disabilities (Hooper et al. 2008). The data were collected under conditions of routine clinical practice, and in NHSTT services, scales may be completed in online formats or with a therapist either face to face or by telephone or video communication (National Collaborating Centre for Mental Health 2024). We do not know the conditions under which the measures used in this paper were completed, although the conditions are typical of the context in which they are used and the limited research in this area suggests that the structure of such scales is consistent across delivery methods (e.g., Ryan et al. 2013). Due to limitations of data linkage processes, we do not have data on levels of disability for the participants, but they are likely to be people with relatively mild intellectual disability, and we cannot generalise these data beyond the populations of people with intellectual disabilities attending NHSTT.

## 5 Conclusions

This paper has described CFA and internal reliabilities of the PHQ‐9, GAD‐7 and WSAS when used with people with intellectual disabilities in an English NHS Talking Therapies for anxiety and depression (NHSTT) service in the north of England. We have reported that the characteristics of the scales when used with people with intellectual disabilities are similar to their characteristics when used with people without intellectual disabilities. The study has some limitations, but the data presented support further exploration of the use of these measures in their original form with people with intellectual disabilities.

## Ethics Statement

The data in this paper were extracted, linked and anonymised for the purpose of evaluation and service improvement and this was the legal basis for processing the data.

## Conflicts of Interest

The authors declare no conflicts of interest.

## Data Availability

The authors have nothing to report.
